# Prognostic models for seizures and epilepsy after stroke, tumors and traumatic brain injury

**DOI:** 10.1016/j.cnp.2025.02.008

**Published:** 2025-03-04

**Authors:** Kai Michael Schubert, Anton Schmick, Miranda Stattmann, Marian Galovic

**Affiliations:** Department of Neurology, Clinical Neuroscience Center, University Hospital and University of Zurich, Zurich, Switzerland

**Keywords:** Prognostic model, Wpilepsy, Post-stroke epilepsy, Post-traumatic epilepsy, Brain tumor-related epilepsy

## Abstract

Epilepsy is a frequent consequence of acute brain injuries, such as stroke, brain tumors, and traumatic brain injury (TBI). Accurate prediction of epilepsy is essential for early intervention and improved patient outcomes. This review evaluates the best-established prognostic models, including the SeLECT and CAVE scores, which estimate the risk of developing seizures and epilepsy following these injuries. The review highlights their clinical applicability, predictive accuracy, and limitations for different etiologies. In addition to providing practical tables for risk estimation, we also offer user-friendly online calculators for these models at www.predictepilepsy.com to facilitate clinical implementation. These tools help identify high-risk patients and support decision-making for follow-up and treatment. Furthermore, we discuss the potential of integrating electrophysiological data, including EEG biomarkers, to further enhance prediction accuracy and patient care. These insights highlight the need for further refinement and validation of predictive models, enabling more personalized treatment strategies and better patient care.

## 1 Introduction

Clinical scoring systems have been foundational in neurology for decades. The Glasgow Coma Scale (GCS), introduced in 1974, revolutionized the systematic assessment of consciousness in brain injury (Teasdale and Jennett, 1974). Earlier, the Hunt and Hess scale (1968) provided a method for grading subarachnoid hemorrhage severity (Hunt and Hess, 1968). The 1980s saw the development of the National Institutes of Health Stroke Scale (NIHSS) for stroke severity (Brott et al., 1989) and the Fisher Scale for predicting vasospasm post-subarachnoid hemorrhage (Fisher et al., 1980). The APACHE II score, established in 1985, included neurological evaluations to predict ICU mortality (Knaus et al., 1985). Additionally, the Glasgow Outcome Scale (GOS), introduced in 1975, has been pivotal in assessing functional outcomes following brain injury (Jennett and Bond, 1975). The modified Rankin Scale (mRS), initially developed in 1957 and later refined, has also become a critical tool for evaluating disability and functional outcomes after stroke (Rankin, 1957).

These scores, based largely on objective measures, have become essential tools in neurology, offering standardized ways to assess patient conditions, predict outcomes, and guide treatment decisions. Their reliability and ease of use have cemented their role in clinical practice and research, enhancing the precision and consistency of neurological evaluations.

In contrast, the use of prognostic models in epileptology has surged only in the past two decades, which is surprising given that epilepsy is one of the most common neurological diseases (Ngugi et al., 2010). Half of adult epilepsy cases are acquired and caused by structural abormalities, such as stroke and traumatic brain injury (TBI), and approximately 60 % of these cases in older adults are due to ischemic or hemorrhagic stroke and brain tumors (Sen et al., 2020). These findings suggest that many epilepsy cases in the elderly have a clear-cut onset, making them good candidates for studying epileptogenesis. This is crucial for developing preventive interventions and novel treatments that target the early mechanisms of epilepsy, potentially halting its progression and improving patient outcomes (Pitkanen and Engel, 2014). This research can also lead to the identification of predictive biomarkers and personalized medicine approaches, ultimately enhancing the quality of life and reducing healthcare costs associated with epilepsy (Pitkanen et al., 2016). However, a significant methodological challenge for anti-epileptogenic drug clinical trials arises from the relatively low probability of post-brain insult epilepsies in the disease population, necessitating the enrichment of patients at risk through the use of predictive scoring systems (Fig. 2).

**Fig. 1:**
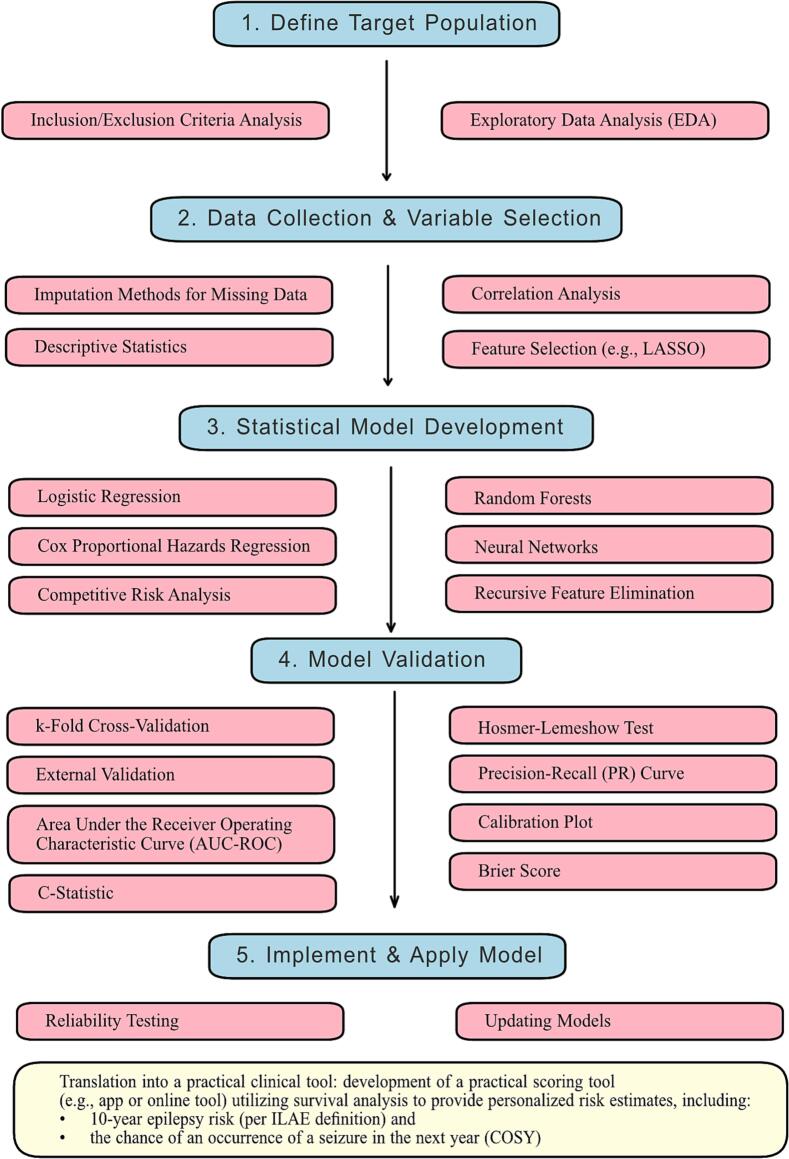
Workflow for Developing, Validating, and Translating Prognostic Models into Practical Clinical Tools.

**Fig. 2:**
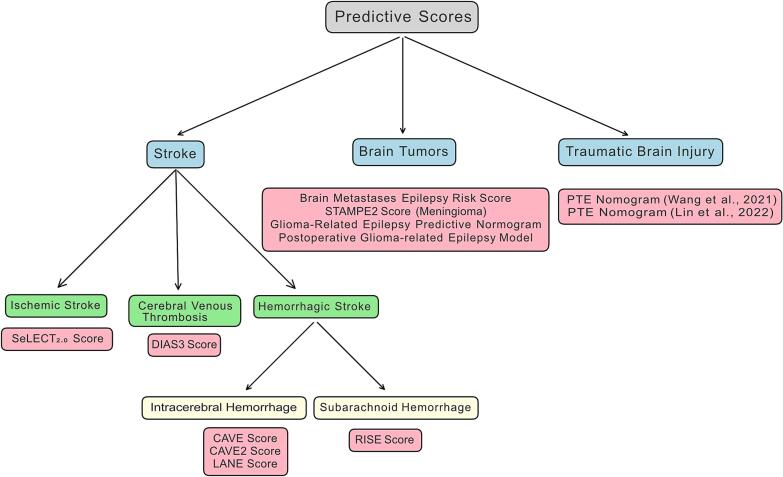
Prognostic models for selzures and epilepsy stroke, tumours and traumatic brain injury.

The objective of this review is to provide a comprehensive overview of existing prognostic models for predicting epilepsy after stroke, tumors and traumatic brain injury, with a particular focus on their clinical applicability, limitations, and the integration of emerging biomarkers, while emphasizing the need to optimize these models to better identify high-risk patients as drug repurposing strategies become increasingly viable for targeted interventions.

## 2 Developing a predictive score for epileptology (Fig. 1 and **Table1**)

### 2.1 Defining the target population

Building a clinical score to predict epilepsy after brain injuries such as stroke, TBI, or intracerebral hemorrhage involves a systematic and comprehensive approach. The process begins with defining the target population, which should include patients with documented cases of stroke, TBI, or intracerebral hemorrhage. Inclusion criteria might encompass adult patients with a confirmed diagnosis of the brain insult through imaging and a specified time frame post-injury. Exclusion criteria may include patients with pre-existing epilepsy or other neurological disorders.

### 2.2 Data collection and Selection of relevant variables

The next step is data collection and selection of relevant variables, which involves gathering clinical and demographic data, imaging findings, neurophysiological findings, and follow-up data. Clinical and demographic data should include age, sex, medical history, type and severity of the brain insult (e.g., Glasgow Coma Scale for TBI, NIH Stroke Scale for stroke), and comorbid conditions (Beghi et al., 2010). Acute symptomatic seizures are defined as those occurring within seven days of a brain insult, such as stroke or traumatic brain injury. This 7-day cutoff helps distinguish these from remote symptomatic seizures, which occur after this period and are indicative of a higher risk for epilepsy (Beghi et al., 2010). However, the 7-day cutoff is rather conventional and has not been independently validated. The time-spectrum for acute symptomatic seizures may be even longer. Imaging findings should focus on MRI or CT scan results, particularly lesion size, location, type (ischemic or hemorrhagic), and cortical involvement (Galovic et al., 2021, Garner et al., 2019, Gupta et al., 2014, Pease et al., 2024). Neurophysiological findings should include EEG data, specifically looking for abnormalities such as epileptiform discharges or electrographic seizures (Bentes et al., 2018, Perucca et al., 2019, Pyrzowski et al., 2024). Additionally, longitudinal follow-up data on the onset of epilepsy, time to first seizure or death, and treatment outcomes are crucial (Sheikh and Jehi, 2024, Steyerberg and Vergouwe, 2014, Yonas et al., 2023).

### 2.3 Statistical methods and model development

Developing the model involves choosing appropriate statistical methods such as logistic regression, Cox proportional hazards models, or machine learning techniques (e.g., random forests, neural networks), depending on the data and the prediction goal (Kuhn and Kjell, 2013; Laupacis et al., 1997, Steyerberg, 2008). Feature selection methods like stepwise regression, LASSO (Least Absolute Shrinkage and Selection Operator), or recursive feature elimination can help in identifying the most predictive variables (Royston et al., 2009, Sanchez-Pinto et al., 2018).

#### Model validation

Validation of the model is essential to ensure its robustness and generalizability. Internal validation can be performed by splitting the dataset into training and validation sets, such as a 70/30 split, and conducting cross-validation, like k-fold cross-validation, to assess model stability and performance (Altman et al., 2009). External validation involves testing the model on an independent dataset from a different cohort (Steyerberg, 2008).

### Assessing model performance: key metrics

#### Discrimination metrics

Discrimination measures the model’s ability to distinguish between patients who will develop epilepsy and those who will not. The area under the receiver operating characteristic curve (AUC-ROC) is a common metric for this purpose (Hanley and McNeil, 1982). However, when dealing with imbalanced class distributions, such as rare events like seizures, the Precision-Recall (PR) curve provides a more informative evaluation. The PR curve focuses on the model's performance in predicting the positive class (e.g., seizure occurrence) and is particularly suited to scenarios where the negative class vastly outnumbers the positive class. It evaluates precision (positive predictive value) against recall (sensitivity), offering complementary insights to the ROC curve.

#### Calibration Metrics

Calibration assesses how well the predicted probabilities match observed probabilities. Calibration plots and the Hosmer-Lemeshow test are standard approaches for evaluating this aspect (Alba et al., 2017; Kuhn, 2013; Steyerberg, 2008).

#### The Brier Score

The Brier score evaluates the overall accuracy of probabilistic predictions by measuring the mean squared difference between predicted probabilities and actual outcomes (Brier, 1950). Unlike discrimination metrics, which focus on the ability to rank predictions correctly, the Brier score incorporates both discrimination and calibration. This makes it a particularly valuable metric for assessing the reliability of a model in probabilistic risk prediction.

#### Implementing and refining the score

Finally, implementing and refining the score involves developing a practical scoring system based on the final model and validating its clinical utility in real-world settings through prospective studies (Kuhn, 2013; Steyerberg, 2008). Conducting reliability testing ensures the score is reproducible, and the model should be regularly updated and refined as more data becomes available and new predictors are identified (Altman et al., 2009).

## 2.4 Practical implementation

In practice, this process would involve gathering comprehensive data from multiple centers, including detailed clinical, imaging, and neurophysiological information on patients with brain injuries. The data should be cleaned and preprocessed to address missing values and outliers. Exploratory data analysis (EDA) helps to understand the distribution and relationships between variables. Feature engineering can create relevant features and normalize or standardize the data as needed. Initial model training could start with logistic regression for simplicity, followed by comparisons with more complex models if necessary. Cross-validation and external datasets should be used for validation (Riley et al., 2024), and model performance should be evaluated using discrimination and calibration metrics. Cross-validation and external datasets should be used for validation, and model performance should be evaluated using discrimination and calibration metrics (Steyerberg and Vergouwe, 2014). The final model should be translated into a practical clinical scoring tool (like an app or online tool) to guide treatment decisions and validated prospectively in clinical settings.

By following these steps and leveraging relevant literature, a robust clinical score for predicting epilepsy risk after brain injuries can be developed, ensuring it is both scientifically sound and practically useful in clinical settings.

## 3 Selected prognostic models for seizures and epilepsy after acute brain injuries (Fig. 2)

### 3.1 Post-Stroke epilepsy

#### 3.1.1 Ischemic stroke

The **SeLECT Score**, is a tool designed to predict the risk of remote symptomatic seizures following ischemic stroke. This model, derived from a cohort of 2,369 patients from four centers (1200 in the derivation and 1169 in the validation cohort), utilizes a multivariable Cox proportional hazards regression. It incorporates five clinical predictors: stroke severity (NIHSS score), large-artery atherosclerotic etiology, acute symptomatic seizures (within 7 days of stroke), cortical involvement, and middle cerebral artery territory involvement. The score assigns points for each predictor, with the total score ranging from 0 to 9. Predictive accuracy was validated with a c-statistic of 0.77. The risk for remote symptomatic seizures ranges from 0.5 % for a score of 0 points to 63.4 % for a score of 9 points within one year (Galovic et al., 2018).

The SeLECT Score underwent further validation and refinement in 2023 with data from the same cohort of 4,552 patients across nine international centers. This updated SeLECT2.0 Score assigns points as follows: NIHSS 4–10 (1 point), NIHSS ≥ 11 (2 points), large-artery atherosclerosis (1 point), short acute symptomatic seizure (3 points), acute symptomatic status epilepticus (7 points), cortical involvement (2 points), and middle cerebral artery territory involvement (1 point). The refined model demonstrated a c-statistic of 0.76, with the risk of remote symptomatic seizures varying from 0.6 % for a score of 0 points to 94 % for a score of 13 points after one year (Sinka et al., 2023).

To aid in driving safety decisions post-stroke, the Chance of Occurrence of Seizure in the next Year (COSY) model was developed using data from the SeLECT2.0 cohort. This model assesses seizure risk to determine safe driving intervals. Patients with SeLECT2.0 scores of 0–6 had a low COSY (0.7 %–11 %), indicating they did not require a seizure-free interval for driving safety. Higher scores necessitated varying seizure-free intervals to reduce COSY to acceptable levels for private and professional driving. Practical tools, such as smartphone-based or web-based applications, have been emphasized for assessing seizure risks and determining appropriate intervals for safe driving (Schubert et al., 2024).

Currently, efforts are underway to integrate EEG findings into the SeLECT Score (SeLECT-EEG) and to develop a separate score for post-stroke survivors with acute symptomatic seizures (SeLECT-ASyS). These developments aim to address the underestimation of long-term risks for post-stroke epilepsy in these groups. Taking into account the NIHSS after possible intervention rather than directly at admission seems to be favorable (Meletti et al., submitted). Additionally, combining the SeLECT score with IL-1β levels has been shown to enhance predictive value for post-stroke epilepsy (Shen et al., 2021).

#### 3.1.2 Hemorrhagic stroke

The ICH-**CAVE Score**, introduced in 2014, predicts epilepsy after intracerebral hemorrhage (ICH). Developed from a derivation cohort of 993 patients in Helsinki, with a validation cohort of 325 patients from Lille, the model includes points for cortical involvement, age under 65 years, hemorrhage volume greater than 10 mL, and acute symptomatic seizures within seven days of ICH. The model's c-statistic was 0.81 in the derivation cohort, indicating strong predictive power, though it dropped to 0.69 in the validation cohort. The risk for remote symptomatic seizures increased with higher scores, ranging from 0.6 % for a score of 0 to 46.2 % for a score of 4 after a median follow-up of 2.7 years (Haapaniemi et al., 2014).

The ERICH study (Kwon et al. 2020) investigated risk factors for seizures following ICH and validated the CAVE score in a multi-ethnic cohort of 2,507 patients. Significant predictors of remote symptomatic seizures included lobar hemorrhage, larger hematoma volume, younger age, and surgical evacuation. The CAVE score showed strong predictive value for seizure development, with an alternative **CAVS score** (substituting surgical evacuation for acute symptomatic seizures) demonstrating similar predictive power (Kwon et al., 2020).

In 2023, the ICH-CAVE Score was refined into the ICH-**CAVE2 Score** using data from 408 patients in Taiwan. This updated model assigns different weights to similar variables: cortical involvement (2 points), age under 65 years (1 point), hemorrhage volume greater than 10 mL (1 point), and acute symptomatic seizures within seven days of ICH (1 point). The c-statistics for the original CAVE and the updated CAVE2 scores were 0.73 and 0.74, respectively. Risk calculations showed a 4.6 % risk of remote symptomatic seizures for scores ≤ 1, 18.3 % for a score of 2, and 54.1 % for scores ≥ 3 over a four-year follow-up (Huang et al., 2023).

Another model, the ICH-**LANE Score**, was developed from a cohort of 602 patients at Qingdao University and validated with 521 patients from Qingdao Municipal Hospital. This model assigns points for lobar hemorrhage, age under 65 years, NIHSS score of 15 or higher, and acute symptomatic seizures. The c-statistic was 0.83 in the derivation cohort and 0.78 in the validation cohort, indicating good predictive accuracy. The risk of remote symptomatic seizures ranged from 0 % for a score of 0 to 100 % for a score of 5 (Y. Wang et al., 2021).

#### 3.1.3 Subarachnoidal bleeding

For subarachnoid bleeding, the SAB-**RISE Score** was developed using a derivation cohort of 419 patients from Vall d'Hebron University Hospital and a validation cohort of 308 patients from Bellvitge University Hospital. Acute symptomatic-onset seizures were observed in 20 % of patients, with epilepsy developing in 12 %. The score includes the modified Rankin scale at onset, ischemia (VASOGRADE system), surgery, and early-onset seizures. The c-statistic was 0.81 in the derivation cohort and 0.86 in the validation cohort. Risk calculations indicated a low risk of 2.9 % for scores 0–1, a moderate risk of 20.8 % for scores 2–3, and a high risk of 75.7 % for scores 4–5 over five years (Campos-Fernandez et al., 2024).

#### 3.1.4 Cerebral venous thrombosis

The **DIAS3 Score** is a predictive tool for estimating epilepsy risk after cerebral venous thrombosis (CVT). It uses six acute-phase clinical variables: decompressive hemicraniectomy, intracerebral hemorrhage at baseline, subdural hematoma, acute seizures (excluding status epilepticus), acute status epilepticus, and age. These variables are combined into a cumulative score that correlates with seizure probabilities over one to three years, ranging from 7 % in low-risk patients to as high as 83 % in high-risk cases. The model was derived from 1,128 patients in the International CVT Consortium and externally validated in two independent multi-center cohorts: ACTION-CVT (543 patients) and the Israel CVT study (556 patients). Across these populations, the DIAS3 score demonstrated robust accuracy, with C-statistics ranging from 0.74 to 0.80, and adequate calibration in both derivation and validation settings. A calculator for the DIAS3 score is available online (Lindgren et al., 2024).

### 3.2 Brain tumor related epilepsy

#### 3.2.1 Meningioma

Focusing on postoperative epilepsy risk in meningioma patients, the Meningioma-**STAMPE2 Score** was developed from a study of 779 patients at the University Hospital Zurich. Pre-surgery, 31 % of patients experienced seizures, and postoperative epilepsy occurred in 26 %. The score includes points for sensorimotor deficits, tumor progression, age under 55 years, major surgical complications, preoperative epilepsy, epileptiform potentials on postoperative EEG, and edema. Treating patients with a score of 2 points or higher is recommended, with prospective validation suggested to guide anti-seizure treatment decisions (Wirsching et al., 2015).

#### 3.2.2 Metastasis

To predict epilepsy risk in patients with brain metastases, particularly focusing on postoperative epilepsy, the **Brain Metastases Epilepsy Risk Score** was developed based on an analysis of 799 patients at the University Hospital Zurich. Of the cohort, 28 % were diagnosed with epilepsy. Points are assigned for supratentorial localization (4 points), incomplete resection (3 points), and multiple surgeries (1 point). The score ranges from 0 to 8, with higher scores indicating a greater risk of postoperative seizures. ROC analysis supported the diagnostic accuracy of the score (AUC = 0.75), suggesting that patients with higher scores might benefit from primary prophylactic anti-seizure medication (ASM) (Wolpert et al., 2020).

#### 3.2.3 Glioma-Related epilepsy predictive models

##### 3.2.3.1 Glioma-Related epilepsy nomogram

The **Glioma-Related Epilepsy Nomogram** predicts the likelihood of postoperative seizures in glioma patients by integrating eight key clinical and molecular factors. These include age, IDH mutation, tumor location (temporal lobe involvement), preoperative epilepsy, extent of resection, WHO grade, functional deficits, and Ki-67 expression levels. Each variable contributes to a cumulative score that reflects seizure risk, with probabilities ranging from 10 % for lower scores to 80 % for higher scores within 12 months following surgery. The nomogram was derived from a cohort of 449 glioma patients stratified by IDH mutation and 1p/19q codeletion status. Although it performed well in this single-center cohort, with clear risk stratification based on clinical and molecular parameters, its generalizability remains limited by its derivation from a single institution and is based on an outdated tumor classification (WHO 2016), which may limit its applicability in present-day clinical settings (Li et al., 2022).

##### 3.2.3.2 Diffuse high-grade gliomas (DHGGs)

A **predictive model for postoperative seizures in patients with diffuse high-grade gliomas (DHGGs)** was developed using clinical and RNA-seq data, identifying a seven-gene signature that significantly improved predictive accuracy. This model, incorporating age, temporal lobe involvement, and preoperative glioma-related epilepsy history, showed strong performance with AUCs of 0.88 and 0.85. Functional analyses pointed to ion channel activities and immune system dysfunctions as key mechanisms in glioma-related epilepsy, highlighting therapeutic targets like SLC1A4 and KCNJ10 (Li et al., 2023).

### 3.3 Traumatic brain injury

A **prognostic model for predicting epilepsy after TBI** was developed using a training cohort of 1,301 patients from West China Hospital (2011–2017) and validation cohorts from Chengdu Shang Jin Nan Fu Hospital (421 patients) and Sichuan Provincial People's Hospital (413 patients). Acute symptomatic seizures were observed in 6 % of the training cohort and 7 % and 5 % in the validation cohorts, respectively. Remote symptomatic seizures occurred in 13 % of the training cohort, 11 % in the first validation cohort, and 6 % in the second. The model incorporates parameters such as sex, time of loss of consciousness, subdural hemorrhage, contusion sites, acute symptomatic post-traumatic seizures, TBI severity, and treatment type. A nomogram was created to provide individualized risk predictions, achieving a c-index of 0.846 in the training cohort, 0.895 in the first validation cohort, and 0.883 in the second validation cohort. This nomogram effectively identifies high-risk individuals, with risk scores for developing epilepsy at 1 and 5 years post-injury ranging from 0 to 100 % (X.-p. Wang et al., 2021).

In contrast, another significant **model for predicting post-traumatic epilepsy after TBI** analyzed 457 patients at Qinghai Provincial People's Hospital from November 2016 to November 2019. This model evaluated high-risk factors for post-traumatic epilepsy by examining different parameters, including contusion site, chronic alcohol use, contusion volume, skull fracture, subdural hematoma (SDH), GCS score, and non-late post-traumatic seizures (Non-LPTS). Significant predictors unique to this model include contusion volumes greater than 13.5 mL, chronic alcohol use, depressed skull fractures, and SDH. The model achieved a c-index of 0.9829, indicating exceptional predictive performance. Internal validation showed strong agreement between predicted and observed outcomes, with the probability of developing post-traumatic epilepsy based on the total score from the nomogram ranging from 1 % for low-risk individuals to over 90 % for high-risk patients (Lin et al., 2022).

Table 2, Wang et al., 2021**and**Table 3 provide a comprehensive overview of the variables and clinical scores used to predict the risk of seizures and epilepsy following stroke, tumors, and traumatic brain injury. Table 2. focuses on the specific variables and their associated risk estimates, while Table 3 summarizes the baseline information, statistical methods, and performance metrics of various prognostic models, highlighting their clinical utility and generalizability. User-friendly online calculators for these models are provided at https://www.predictepilepsy.com to facilitate clinical implementation.

**Table 1:** Commonly Used Statistical Metrics and Techniques in Predictive Modeling.

**Statistical Metric/Technique**	**Context in Predictive Scores**	**Use**	**Limitations**
**Inclusion/Exclusion Criteria Analysis**	Defines the population most relevant to predicting epilepsy risk, ensuring homogeneity in the dataset.	Ensures that the predictive score focuses on appropriate patient groups for reliability.	Overly strict criteria may exclude borderline cases that could reduce generilzabilty and influence predictions.
**Descriptive Statistics of the study cohort**	Summarizes demographic and clinical characteristics of the study population.	Provides insights into the baseline characteristics of the population (e.g.: sex, age,comorbiditie etc.)	Provides a snapshot of the population characteristics without information on causation
**Exploratory Data Analysis (EDA)**	Visualizes and analyzes the data to identify patterns, trends, and relationships.	Useful for detecting missing data, outliers, and trends in variables related to epilepsy risk.	Subjective interpretation may introduce bias; requires statistical validation of observed trends.
**Imputation Methods for Missing Data**	Fills gaps in datasets where variable values are missing due to incomplete data collection.	Prevents loss of valuable information and allows the use of the full dataset.	Imputed data may not accurately reflect real-world conditions and could bias the model.
**Correlation Analysis**	Examines relationships between variables (e.g., lesion size and seizure risk) to identify potential predictors.	Helps in selecting variables strongly associated with epilepsy risk.	Cannot capture non-linear relationships; correlation does not imply causation.
**Feature Selection (e.g., LASSO)**	Identifies the most predictive variables by penalizing irrelevant ones, reducing model complexity.	Prevents overfitting and improves generalizability of the predictive model.	May exclude important variables in noisy or highly correlated datasets.
**Logistic Regression**	Used for binary classification problems, such as predicting the presence or absence of epilepsy.	Provides interpretable coefficients and estimates the probability of outcomes.	Assumes linear relationships between predictors and the log odds, which may oversimplify complex data.
**Cox Proportional Hazards Regression**	Models time-to-event data, such as the time until epilepsy onset after brain injury.	Identifies time-dependent risk factors for epilepsy and provides hazard ratios.	Assumes proportional hazards over time, which may not hold true in all datasets.
**Competitive Risk Analysis**	Analyzes the likelihood of specific events (e.g., epilepsy vs. death) in the presence of competing risks.	Provides more realistic modeling for datasets with multiple possible outcomes.	Requires careful handling of censored data; complexity increases with the number of competing events.
**Random Forests**	A machine learning technique that uses an ensemble of decision trees to model complex relationships between variables.	Handles large datasets with many predictors and captures non-linear interactions.	Prone to overfitting without careful parameter tuning; limited interpretability. In cases of linear correlation they may not enhance precision as compared to a single tree.
**Neural Networks**	Used for detecting complex patterns in data, mimicking the human brain’s processing abilities.	Effective for modeling non-linear and multi-dimensional relationships.	Requires large datasets and high computational power; prone to overfitting if not properly regularized. Risk of data set bias.
**k-Fold Cross-Validation**	Splits the dataset into k subsets to repeatedly train and test the model, ensuring robustness.	Provides a reliable estimate of model performance and prevents overfitting.	Computationally intensive with large datasets; results depend on the number of folds chosen.
**Recursive Feature Elimination**	Repeatedly construcs a model and removes less important predictors to identify the most impactful variables.	Improves model simplicity and identifies key variables influencing the outcome.	Can be computationally expensive; may miss subtle interactions between predictors.
**Area Under the Receiver Operating Characteristic Curve (AUC-ROC)**	Measures the model’s ability to distinguish between those who develop epilepsy and those who do not.	Commonly used to evaluate the discriminatory power of the model.	Limited to binary outcomes. Does not assess model’s precision and negative predictive value. Can be misleading for imbalanced datasets; a high AUC may have low precision and negative predictive value.
**Z Score**	Standardizes raw scores by centering data around the mean and scaling by the standard deviation, enabling scores from different distributions to be compared directly.	Facilitates direct comparisons between different datasets or models, aids in outlier detection, and improves model interpretability.	Assumes a roughly normal distribution; sensitive to outliers and may be misleading if the underlying data distribution deviates significantly from normal.
**Precision-Recall (PR) Curve**	Evaluates the model’s performance in predicting the positive class (e.g., seizure occurrence), particularly in imbalanced datasets.	Highlights precision (positive predictive value) versus recall (sensitivity).	Interpretation varies with class imbalance; does not account for performance on the negative class.
**Calibration Plot**	Visualizes how well the predicted probabilities align with observed outcomes in real-world applications.	Identifies overconfidence or underconfidence in model predictions during practical use.	Requires manual interpretation and does not provide a numeric measure of calibration quality.
**Hosmer-Lemeshow Test**	Statistically evaluates the goodness-of-fit between observed and predicted probabilities during implementation.	Confirms the reliability of the model’s calibration numerically.	Sensitive to sample size; results may be misleading in small or overly large datasets. Thus, no longer recommended.
**Brier Score**	Combines discrimination and calibration to evaluate the overall accuracy of probabilistic predictions.	Provides a single metric to evaluate both the reliability and accuracy of predictions.	Averages performance over all probabilities. It has a poor performance in very rare or very frequent events due to minor accountability of small changes.
**C-Statistic**	Measures the concordance between predicted risks and observed outcomes, similar to AUC-ROC.	Indicates the precision of risk predictions, commonly used in survival analysis models.	Does not provide specific insights into either discrimination or calibration.
**External Validation**	Evaluates the model’s performance on an independent dataset from a different cohort or population.	Confirms the generalizability of the model beyond the training data.	Requires access to external datasets; differences between datasets may result in biased evaluations.
**Reliability Testing**	Assesses reproducibility of the model’s predictions across different subsets of the population.	Ensures the model performs consistently in diverse real-world settings.	Requires repeated testing in different clinical settings, resource-intensive.
**Updating Models**	Refines the predictive model as new data becomes available, ensuring it remains relevant over time.	Adapts the model to changing clinical practices or populations.	Requires continuous monitoring and data collection; risk of introducing errors during updates.

**Table 2:** Provides a practical chart with variables for the calculation of risk estimates to predict seizures and epilepsy following stroke, tumors, and traumatic brain injury. The table outlines key clinical factors, each assigned specific point values, that contribute to a patient's overall risk score. These scores are then used to estimate the likelihood of developing seizures over various time frames, such as two or five years. The chart is designed to guide clinicians in assessing patient risk and making informed decisions about monitoring and treatment based on individualized risk profiles.

**Table 3:** Summarises baseline information and the quality of various prognostic models used to predict seizures and epilepsy following stroke, tumors, and traumatic brain injury. these models, derived from diverse patient populations, employ statistical methods like cox regression to assess seizure risk. each model’s performance is evaluated using metrics such as c-statistics and auc, which indicate their predictive accuracy. the models vary in generalizability, with some validated across multiple centers, while others are limited to single-center studies. clinically, these models are valuable for guiding treatment decisions, monitoring, and follow-up in patients at risk for seizures.

**Score Name**	**Population**	**Statistical Methods**	**Validation Methods**	**Performance Metrics**	**Generalizability**	**Clinical Utility**
**SeLECT_2.0_ Score** **(**Galovic et al., 2018, Sinka et al., 2023**)**	2023: Derivation: 4552 patients, 9 international centers (2002–2019)	Multivariable Cox proportional hazards regression	Internal: Derivation cohort (9 international sub-cohorts); 2023 Replication cohort for Status epilepticus (SE): 39 patients with post-stroke SE, 3 separate cohorts	C-statistic: 0.77 (derivation), Optimism-corrected discrimination: 0.77 Risk scores: 2 % (0 points) to 100 % (13 points)	High (multicenter validation)	High (assists in guiding treatment and follow-up), mobile App available (https://predictapps.github.io/select/)
**CAVE Score** **(**Haapaniemi et al., 2014**)**	Derivation: 993 patients (n = 764 > 7 days survivors), Helsinki (2005–2010)Validation: 325 patients, Lille (2004–2009)	Cox regression model	Internal: Retrospective cohort (University Hospital Helsinki) External: 325 patients, Lille (2004–2009)	c-statistic: 0.81 (derivation), 0.69 (validation) Risk scores: 0.6 % (0 points) to 46.2 % (4 points)	Moderate to high (lower validation c-statistic)	Good (simple, easy to use)
**CAVE2 Score** **(**Huang et al., 2023**)**	Derivation: 408 patients, Taiwan (2013–2019)	Binary logistic regression	Internal: Retrospective cohort (Ditmanson Medical Foundation Chiayi Christian Hospital)	c-statistic: 0.74 Risk scores: 4.6 % (≤1 point), 18.3 % (2 points), 54.1 % (≥3 points)	Moderate (limited by single-center study)	Good (improved accuracy over CAVE score)
**LANE Score** **(**Wang et al., 2021**)**	Derivation: 602 patients, Hospital of Qingdao University (2014–2017)Validation: 521 patients, Qingdao Municipal Hospital (2015–2017)	Cox regression model	Internal: Retrospective cohort (Affiliated Hospital of Qingdao University) External: 521 patients, Qingdao Municipal Hospital	c-statistic: 0.83 (derivation), 0.78 (validation) Risk scores: 10.1 % (0 points) to 100 % (6 points)	High (validated in a separate cohort)	Good (useful for closer monitoring and clinical trials)
**RISE Score** **(**Campos-Fernandez et al., 2024**)**	Derivation: 419 patients, Vall d’Hebron University Hospital (2012–2021)Validation: 308 patients, Bellvitge University Hospital (2011–2022)	Multiple Cox regression model	Internal: Retrospective cohort (Vall d’Hebron University Hospital) External: 308 patients, Bellvitge University Hospital	AUC: 0.82 (derivation), 0.82 (validation) Risk scores: 2.9 % (0–1 points), 20.8 % (2–3 points), 75.7 % (4–5 points)	High (validated in a separate cohort)	High (guides personalized treatment and follow-up)
**Brain Metastases Epilpsy Risk Score** **(**Wolpert et al., 2020**)**	Derivation: 799 patients, Zurich (2004–2014)	Univariate and multivariate Cox regression models	Internal: Retrospective cohort (University Hospital Zurich)	AUC: 0.75 (derivation) Risk scores: 0 % (0 points) to 47 % (8 points)	Moderate (limited by retrospective design)	Fair (helps identify high-risk patients for ASM treatment and/or potential prophylaxis)
**STAMPE2 Score** **(Wirsching et al., 2016)**	Derivation: 779 patients, Zurich (2000–2013)	Binary logistic regression model	Internal: Retrospective cohort (University Hospital Zurich)	OR for each variable provided: e.g., Preoperative epilepsy OR: 3.46 (95 % CI: 2.32–5.16), CNS infection OR: 5.89 (95 % CI: 1.53–22.61)	Moderate (limited by retrospective design)	Fair to good (guides postoperative ASM treatment but without specifying risk in percentages and follow-up periods)
**Postoperative Glioma-related Epilepsy Model** **(**Li et al., 2023**)**	Derivation: 166 patients, Validation: 42 patients, Beijing Tiantan Hospital	LASSO logistic regression, multivariate logistic regression	Internal: Retrospective cohort (Beijing Tiantan Hospital)	AUC: 0.878 (training), 0.845 (validation)	Moderate (single-center study)	Fair to good (combines gene-signature and clinical data), though the complex score with the nomogram is challenging to apply in practice, it effectively identifies high-risk patients
**Glioma-Related Epilepsy Predictive Normogram (Li et al., 2021)**	449 patients stratified by IDH mutation and 1p/19q codeletion status.	Multivariate logistic regression and Cox proportional hazards regression.	Retrospective cohort, single-center study.	No AUC or c-statistics reported. Performance is evaluated using odds ratios for specific factors.	Moderate (limited to single-center cohort, specific to glioma-related epilepsy).	Guides AED use and seizure management strategies in glioma patients.
**PTE Nomogram (1)** **(**Wang et al., 2021**)**	Derivation: 1301 patients, West China Hospital (2011–2017) Validation: 834 patients, two cohorts (2013–2015)	Multivariable Cox proportional hazards regression model	Internal: Retrospective cohort (West China Hospital) External: Two independent cohorts (421 patients from Chengdu Shang Jin Nan Fu Hospital and 413 patients from Sichuan Provincial People’s Hospital)	C-index: 0.846 (training), 0.895 (validation)	High (validated in two separate cohorts)	Good to high (guides targeted treatment and follow-up)
**PTE Prediction Nomogram (2)** **(**Lin et al., 2022**)**	Derivation: 457 patients, Qinghai Provincial People’s Hospital (2016–2019)	Univariate and multivariate logistic regression	Internal: Retrospective cohort (Qinghai Provincial People’s Hospital)	C-index: 0.9829 Risk scores: 1 % (131 points) to 99 % (332 points)	Moderate (single-center study)	Fair (identifies high-risk patients for targeted ASM treatment and/or prophylaxis)
**DIAS3 Score** **(**Lindgren et al., 2024**)**	Derivation cohort: 1128 patients (International CVT Consortium), validation cohorts: 543 (ACTION-CVT) and 556 (Israel CVT study). Multinational, hospital-based.	Cox proportional hazards regression with multiple imputation and ridge penalty adjustments for overfitting	Internal validation with bootstrapping, external validation using two independent multicenter cohorts	C-statistics: 1-year and 3-year follow-up – derivation cohort (0.74), ACTION-CVT (0.76, 0.77), Israel CVT cohort (0.80). Calibration plots indicated adequate agreement	High (validated internationally across multiple centers with diverse patient cohorts)	Good (provides personalized epilepsy risk estimates after CVT, but only at 1 and 3 years, offers an online calculator https://cerebralvenousthrombosis.com/professionals/dias-3/)

Legend: Score Name refers to the name and reference; Population describes the patient data used; Statistical Methods outlines the model development approach; Validation Methods explain how the scores were tested; Performance Metrics indicate predictive accuracy; Generalizability shows the applicability across different settings; Clinical Utility reflects the practical use of these scores in patient care.

Abbreviations: C-statistic (Concordance statistic), AUC (Area Under the Curve), ASM (Antiseizure medication), OR (Odds Ratio), LASSO (Least Absolute Shrinkage and Selection Operator), CNS (Central Nervous System), CI (Confidence Interval).

### 3.4 Advantages and disadvantages of simple scores and complex nomograms in predicting epilepsy after brain injuries

When developing clinical prediction models for epilepsy after brain injuries, such as stroke or TBI, both simple scores and complex nomograms have unique advantages and disadvantages, particularly regarding the risk of overfitting.

Advantages of Simple Scores: (Steyerberg and Vergouwe, 2014).

• **Ease of Use:** Simple scores like the CAVE or SeLECT scores offer straightforward calculations that can be quickly applied in clinical settings without the need for advanced technology.
• **Rapid Decision-Making:** These models facilitate quick risk stratification at the bedside, enabling timely interventions.
• **High Compliance:** Due to their simplicity, they are more likely to be used consistently by healthcare providers.
• **Accessibility:** Simple scores are particularly valuable in resource-limited settings where access to advanced computational tools may be limited.
• **Broad Applicability:** These scores can be applied across various clinical settings with minimal adaptation.

#### Disadvantages of simple Scores

• **Underfitting:** The simplicity of these models may lead to the omission of important interactions and nuances, potentially reducing their predictive power and precision.
• **Limited Personalization:** They may not provide highly individualized risk assessments, which can be crucial for personalized medicine.

#### Advantages of complex Nomograms: (Jimenez et al., 2022)

• **High Predictive Accuracy:** Complex models incorporate a larger number of variables and can model intricate interactions, often resulting in higher c-statistics and more accurate predictions.
• **Personalized Medicine:** These models align with the principles of personalized medicine by providing highly individualized risk assessments.
• **Detailed Risk Stratification:** They can capture detailed patient-specific information, which can be vital for nuanced clinical decision-making.

#### Disadvantages of complex Nomograms

• **Risk of Overfitting:** The complexity of these models introduces a higher risk of capturing noise rather than true underlying patterns, which can lead to poor generalizability in new datasets.
• **Resource Intensive:** They require advanced computational tools and extensive training, which can limit their practical application in low-resource environments.
• **Implementation Barriers:** The complexity and need for regular updates and validation can pose challenges in clinical implementation.

**Balancing Simplicity and Complexity:** To mitigate overfitting in simple models, techniques such as cross-validation and focusing on well-validated clinical variables are essential. For complex models, regularization methods like LASSO, extensive cross-validation, pruning of non-contributory variables, and external validation with independent cohorts are crucial. By balancing these factors, robust, reliable, and clinically useful prediction tools can be developed.

## 4 Integrating electrophysiological data in seizure prediction

### 4.1 The necessity of integrating electrophysiological data into prediction scores for post-stroke epilepsy

Epilepsy is fundamentally an electrophysiological disorder. Despite EEG's sensitivity in detecting acute cerebral ischemia(Jordan, 2004) and evaluating brain function (Assenza and Di Lazzaro, 2015), its role in predicting post-stroke epilepsy remains underexplored. Patient self-reporting is unreliable and may affect prediction models, leading to potential discrepancies between predicted and observed outcomes. For example, seizures originating from or involving the left hemisphere are often of an amnestic nature, where patients may not remember the event and fail to report it to their doctor (Lux et al., 2002). It is surprising that electrographic findings and EEG biomarkers have not received much attention in current prediction scores, which primarily focus on seizure occurrence as the outcome. Typically, these scores are retrospectively collected, emphasizing seizure occurrence over electrographic data. Furthermore, it should be noted that studies investigating EEG biomarkers have predominantly relied on standard 20-minute EEG recordings.

Significant predictors of postoperative epilepsy in patients with brain tumors, such as meningiomas, include the presence of epileptiform potentials on EEG. Specifically, preoperative epileptic discharges and postoperative focal slowing have been linked with persistent epilepsy following surgical resection of meningiomas (Wirsching et al., 2015). One study analyzed EEG data for pre- and postoperative epilepsy in brain metastases patients, but these findings were underutilized in the scoring model, being mentioned only to justify AED prophylaxis in 30 patients (22 %) (Wolpert et al., 2020).

Studies on post-ischemic and post-hemorrhagic stroke patients have demonstrated that integrating electrographic findings into prediction scores could significantly enhance the prediction of post-brain insult epilepsy. This is particularly beneficial for lower-risk groups—patients without acute symptomatic seizures, severe comorbidities, or large brain injuries—who represent the largest demographic developing post-stroke epilepsy in the long run. Up to 12 % and 25 % of acute ischemic stroke patients present with electrographic seizures (ESz) and rhythmic or periodic patterns (RPPs) on EEG during the acute phase (Belcastro et al., 2014, Scoppettuolo et al., 2019). The risk factors for ESz and RPPs align with those for the development of post-stroke epilepsy, as mentioned above, suggesting they may herald post-stroke epilepsy (Bentes et al., 2018).

Similarly, in TBI contexts, EEG biomarkers show promise in predicting post-traumatic epilepsy. Key EEG abnormalities, such as interictal epileptiform discharges (IEDs), lateralized periodic discharges (LPDs, formerly PLEDs), and generalized periodic discharges (GPDs, formerly GPEDs), are consistently associated with increased post-traumatic epilepsy risk (Appavu et al., 2022, Chen et al., 2023, Tubi et al., 2019). Focal slowing and lateralized rhythmic delta activity, especially when assessed through continuous EEG (cEEG, usually > 12 h) monitoring in the acute phase post-TBI, also link to higher post-traumatic epilepsy risk (Kim et al., 2018, Pease et al., 2023).

In pediatric and young adult populations, sleep abnormalities such as absent or diminished spindles over one hemisphere have emerged as additional predictors (Appavu et al., 2022). These findings highlight the importance of considering both epileptiform activity and background EEG abnormalities, including sleep disruptions, in predictive models.

Continuous EEG monitoring has advanced the field of electrographic analysis. Prolonged long-term EEG (LT-EEG) monitoring, particularly during sleep, has demonstrated a significantly higher yield in detecting interictal epileptiform discharges (IEDs) compared to standard EEG De Stefano et al., 2023. For post-stroke epilepsy, highly epileptogenic rhythmic and periodic patterns correlate with post-stroke epilepsy development and can complement clinical risk factors (Punia et al., 2022, Tatillo et al., 2024). Given EEG's high sensitivity in detecting acute cerebral ischemia and assessing brain function, incorporating EEG data into prediction models could enhance their accuracy. Electrographic biomarkers on standard, commonly used (usually 20–60 min), and clinically available EEG, as well as continuous EEG during the acute phase of ischemic stroke, associate with higher post-stroke epilepsy risk. These EEG findings provide complementary prognostic information to clinical risk factors, helping identify patients who might benefit most from short or continuous EEG recording. Emerging quantitative EEG algorithms may further enhance the predictive power of EEG biomarkers for post-brain insult epilepsy.

In conclusion, integrating electrophysiological data into prediction scores for post-brain insult epilepsy, including post-stroke, post-traumatic, and brain tumor-associated epilepsy, could markedly improve their predictive power. Due to their high sensitivity and association with cortical injury, EEG biomarkers should be considered in future prognostic models. This approach could be especially beneficial for identifying lower-risk patients who may otherwise go unnoticed by current clinical scores, enabling more tailored and effective management strategies for stroke and TBI survivors.

## 5 Current advances in antiepileptogenesis studies following acute brain injuries in adults

Until now, no medication has been definitively proven to influence epileptogenesis, though recent evidence suggests that certain drugs may hold potential. While ASMs are known to affect mortality, their role in preventing epilepsy is less clear. For example, a recent study found that initiating ASM within 48 h of a first seizure significantly improved long-term outcomes, with a lower recurrence rate observed in those treated early (Menetre et al., 2024). Animal studies have indicated antiepileptogenic properties in drugs such as levetiracetam, brivaracetam, eslicarbazepine, and topiramate, with levetiracetam being the most systematically investigated for post-traumatic epilepsy (Klein et al., 2018, Klein et al., 2020). However, a recent study on eslicarbazepine showed no significant effect, only a nominal reduction in unprovoked seizures by approximately 50 % f(Koepp et al., 2023) Further research into perampanel is ongoing Nicolo et al., 2021.

In addition to ASMs, other medications have shown potential for antiepileptogenesis. For instance, angiotensin receptor blockers (ARBs) have been associated with a decreased incidence of epilepsy in hypertensive patients. A large cohort study in Germany involving 168,612 patients found that ARB therapy was linked to a significantly lower incidence of epilepsy compared to other antihypertensive drugs, suggesting ARBs like losartan could be a novel approach for epilepsy prevention (Doege et al., 2022). This finding was further supported by a study involving over 2 million U.S. patients, which confirmed the antiepileptogenic effect of ARBs even after accounting for cerebrovascular incidents (Wen et al., 2024). Additionally, a population-based study in Taiwan showed that both ARBs and ACE inhibitors significantly reduced the risk of poststroke epilepsy, with the greatest benefit observed in younger patients who continued their therapy after a stroke (Chang et al., 2024).

Similarly, statins have shown promise in reducing the risk of developing epilepsy after brain injuries. A critical review highlighted the potential of statins, particularly atorvastatin, in lowering epilepsy risk (Hufthy et al., 2022). Moreover, newer glucose-lowering drugs (GLDs) have demonstrated neuroprotective and anti-inflammatory properties, suggesting they might influence seizure and epilepsy development (Citraro et al., 2019, Kopp et al., 2022, Liu et al., 2018).

While only a small percentage of individuals develop epilepsy following ischemic or hemorrhagic stroke or TBI (3–12 %), this extensive body of research underscores the need for robust, accurate, and generalizable models to predict epileptogenesis after brain injuries (Galovic et al., 2021, Pease et al., 2024). Such predictive models could correct for confounding factors and support the enriched recruitment of high-risk patients in prospective trials. This approach is crucial for advancing the development of preventive strategies and ultimately improving patient outcomes in the field of epilepsy.

## 6 Conclusion

Predictive scores have long served as a cornerstone in neurology, significantly shaping clinical practices and enhancing patient outcomes. In the field of epileptology, however, their critical role has only recently gained recognition, particularly in the management of epilepsy following acute brain injuries such as stroke and TBI. These tools are now understood to be vital for stratifying patients, informing their families, and planning follow-up diagnostics and interventions, ultimately influencing the long-term care strategy. Table 2. and Table 3 provide a detailed comparison of key scores. To further support clinical implementation, we have developed user-friendly online calculators for these models, available at https://www.predictepilepsy.com.

The development of reliable predictive scores is not only crucial for advancing antiepileptogenesis studies – which aim to prevent the onset and progression of epilepsy – but also for practical clinical applications. These include informing therapeutic decisions, guiding patient monitoring, and identifying those who might benefit most from early interventions. Established scoring systems, such as the SeLECT Score for ischemic stroke and the ICH-CAVE Score for intracerebral hemorrhage, offer structured approaches to identifying patients at high risk of remote symptomatic seizures. These models utilize clinical predictors like stroke severity, cortical involvement, and acute symptomatic seizure occurrence, providing a framework that could be further refined with the integration of electrophysiological data such as EEG findings.

EEG biomarkers, which have demonstrated potential in identifying high-risk patients, complement clinical risk factors and aid in better patient stratification and the customization of interventions. The ongoing advancements in continuous EEG monitoring and emerging quantitative EEG algorithms enhance our ability to analyze electrographic patterns linked to the development of post-stroke epilepsy and post-traumatic epilepsy. These insights underscore the necessity of incorporating EEG data into prediction models to improve their predictive accuracy. Beyond EEG biomarkers, future prognostic models could benefit from integrating other biomarkers, such as genetic, proteomic, and metabolomic data.

Despite their promise, several barriers hinder the widespread adoption of these models in clinical practice. One significant challenge is the need for further validation and integration of these tools into routine clinical workflows. For example, while some predictive models have undergone external validation, data on their performance in diverse populations, particularly across different ethnic groups, remain sparse. Moreover, while these models can inform risk assessment, their direct application in therapeutic decision-making remains limited. To bridge this gap, continuous efforts are required to refine these models, ensuring they are robust and user-friendly for clinicians.

Looking to the future, the integration of artificial intelligence (AI) and machine learning holds immense potential for revolutionizing predictive modeling in neurology. AI is particularly powerful in analyzing high-dimensional data or long-term datasets, such as those from MRI, continuous EEG monitoring, or emerging biomarkers like genetic, proteomic, and metabolomic data. These models excel at processing complex data, identifying subtle patterns, and refining predictive algorithms, which is essential for personalized medicine approaches. By leveraging AI, we can not only enhance the predictive accuracy of these models but also enable their application in real-time clinical settings, thereby improving patient care. While AI holds great potential to transform prediction models, challenges such as overfitting and the black-box nature of machine learning algorithms remain significant. Overfitting can result in poor generalization to external datasets, while lack of interpretability may hinder clinical trust. Ensuring transparency, explainability, and rigorous external validation is essential for the successful integration of AI-driven models into clinical practice.

In addition to the optimization, validation, and refinement of predictive models in larger datasets and the definition of specific subgroups, other underrepresented areas require focused research. Certain age groups, such as pediatric and elderly populations, present unique clinical trajectories and risk factors that demand tailored models. For rarer etiologies, including autoimmune encephalitis and various brain tumors (e.g., lymphoma), predictive models remain underexplored. In other areas, such as metastatic brain tumors, updates to predictive models are urgently needed, as therapeutic advancements, including checkpoint inhibitors and CAR T-cell therapies, have significantly transformed treatment possibilities. These examples reflect the manifold opportunities to advance predictive modeling for a broader range of clinical scenarios.

In conclusion, the ongoing refinement of predictive scores, combined with the integration of electrophysiological and advanced computational methods, holds immense potential for advancing antiepileptogenesis research. These developments are pivotal not only for identifying predictive biomarkers but also for implementing personalized medicine approaches that improve patient outcomes, inform therapeutic decisions, and reduce the burden of epilepsy on individuals, families, and healthcare systems.

**Funding**

The author(s) received no financial support for the research, authorship, and/or publication of this article. MG received fees and travel support from Arvelle, Advisis, Bial and Nestlé Health Science outside the submitted work.

## Data Availability

Data will be made available on request.
